# Audiovestibular and vaccination complications of COVID-19

**DOI:** 10.1186/s43163-022-00290-2

**Published:** 2022-08-19

**Authors:** Takwa Gabr, Mona Kotait, Asmaa Salah Moaty

**Affiliations:** 1 Audiovestibular Medicine Unit, Otolaryngology Department, Kafrelsheikh University Hospitals, Kafr Elsheikh, Egypt; 2 Audiovestibular Medicine Unit, Otolaryngology Department, Tanta University Hospitals, Tanta, Egypt; 3 Audiovestibular Medicine Unit, Otolaryngology Department, Shebin Elkom University Hospitals, Shebin Elkom, Egypt

**Keywords:** COVID-19, Coronavirus infections, Hearing loss, Vestibular diseases, COVID-19 vaccines

## Abstract

**Objectives:**

Since its first appearance in Wuhan December 2019, SARS-CoV2 virus received great attention due to its severe symptoms and high spread causing COVID-19 disease which spread all over the world like a pandemic. The causative virus is capable of human-to-human transmission via droplet and direct contact suggesting that upper respiratory tract is the main site to virus manifestations.

There is a great diversity in its clinical picture, although the severe respiratory and neurological symptoms are commonly present; however, other symptoms are present. Although otological manifestations are reported in many COVID-19 patients even in asymptomatic cases, they did not receive much attention compared with other critical manifestations. In this article, we paid our attention specifically to the otological manifestations of COVID-19 and their relevance either to the virus infection, treatment, or vaccination through literature review.

**Conclusion:**

COVID-19 disease has a deleterious effect on the inner ear. This effect is not only due to SARS-Cov-2 infection, but it could be also due to the ototoxic drugs used for treatment. The COVID-19 vaccinations are found to be implicated in the otological symptoms in some cases.

## Introduction

Coronavirus disease 2019 (COVID-19) is a contagious respiratory and vascular disease that was found to be caused by severe acute respiratory syndrome coronavirus 2 (SARS-CoV-2 virus). Patients of COVID-19 are presented with many symptoms and signs with great variability in their clinical pictures, and clinical findings were noticed worldwide. However, the most common symptoms are fever, shortness of breath, cough, sore throat, headache, muscle pain, and taste and smell disturbance. The incidence of otological symptoms is quite small including hearing loss, vestibular symptoms, tinnitus, and otitis media in addition to rare symptoms as facial palsy or migraine. Some asymptomatic cases with positive polymerase chain reaction (PCR) for COVID-19 showed evidence of inner ear affection. This might indicate that the prevalence of otological signs of auditory damage related to SARS-CoV-2 infection is much more than we thought. In this review, we tried to highlight the otological manifestations reported in different studies and their relevance to treatment or vaccination.

## Method

In this review, English-language studies were searched in PubMed and the Cochrane databases about COVID-19 published in 2019, 2020, and 2021. These studies included meta-analyses, systematic reviews, randomized clinical trials (RCTs), practical guidelines, and case reports. The references of the selected articles were searched for additional data.

## Discussion

By the end of 2019, a new coronaviruses family member appeared in Wuhan-China where several cases of pneumonia appeared with unidentified etiology. The new coronavirus was identified on 6 January 2020 as the cause of these cases and was named as SARS-CoV-2, and on 30 January 2020, WHO declared the coronavirus disease as COVID-19 and declared the pandemic state with public health emergency of international concern [1]. The disease started with symptoms of viral pneumonia, including fever, cough in addition to dyspnea, and bilateral lung infiltration in severe cases [2]. In general, SARS-CoV-2 (COVID-19) viruses single-stranded RNA that have a close sequence similar to bat coronaviruses which appeared to be the primary source. Like other viruses, SARS-CoV-2 evolves over time and several variants had emerged which were designated in Greek labels by the WHO: alpha, delta, beta, gama, and omicron [3].

### Infection

SARS CoV-2 virus can be found in the saliva, tears, and cerumen of both symptomatic and asymptomatic patients. Moreover, Hanege et al. [4] reported the possibility of ocular transmission. Some authors suggest that nasolacrimal duct might be responsible for the access of the virus to the conjunctiva of the infected individuals causing conjunctivitis. Hanege et al. [4] demonstrated that around 40% of the COVID-19 patients had detectable amounts of SARS-CoV-2 genome in their cerumen with possibility of viral transmission via the patient’s hands while scratching their ears.

Initially, it was suggested that the SARS CoV-2 virus spread from animals to human in Wuhan market. However, the possibility of human-to-human can occur through the close contact with an infected person, exposure to coughing, sneezing, respiratory droplets, or aerosols which can penetrate the human lungs via inhalation through the nose or mouth [5].

The main inter-human infection pathway is the projection of droplets coming from lower and upper airways which remain stable for more than 72 h on many surfaces. Exposure to infection with COVID-19 in the otolaryngology field is mainly through exposure to infection from anatomical areas where the virus is highly concentrated, mainly in the nasal fossa and nasopharynx even in asymptomatic patients [6].

The virus remains alive in the air with a greater risk of being transmitted by aerosols with high concentration in relatively closed environment. The dissolution of the virus in an aerosol forms a bio-aerosol which help the spread of infection for several meters and for 3 hours [7]. Many physiological procedures (coughing, sneezing) and mechanical procedures (intubation, endoscopy, noninvasive ventilation) produce aerosols. Even the use of high-speed drills in mastoidectomy carry great risks of generating aerosols of the bone, mucosa, fluids, or blood that could be dangerous for people inside the operating room to viral infection [8].

### Pathology of the virus

The genome of SARS-CoV-2 encodes for a nuclear protein (N-protein) that allows the virus to attack human cells and turned them into virus factories. The N protein plays a vital role in virus replication and transcription. Another type of protein “S-protein” is also integrated over the surface of the virus and is responsible for the attachment of the virus to the host cell surface receptors to facilitate viral entry into the host cell [9]. Additionally, the envelop protein (E-protein) is a small membrane protein that plays an important role in membrane permeability of the host cell and virus-host cell interaction [10].

*The coronavirus spike (S) protein facilitates* viral entry into target cells depending on the binding of the surface unit of the S protein (S1) to the cellular receptors (angiotensin converting enzyme 2, ACE2) found on the surface of many human cells, including those in the lungs, heart, kidney, and skin as well. Virus entry also requires S protein priming by cellular proteases (transmembrane protease serine 2 (TMPRSS2) with the release of the fusion peptide that triggers the activation of the membrane fusion mechanism [11]. Once the SARS-Cov-2 entered the cytoplasm, it induces membrane fusion followed by the release of the virus genetic material (a single-stranded RNA) into the cytoplasm where the replication and transcription of viral RNA. Viral proteins are then formed and called virions which are exported from the infected cells through exocytosis to infect other cells [12].

### Pathophysiology of audiovestibular manifestations of COVID-19

The exact pathophysiology of audio-vestibular manifestations of COVID-19 is unknown. The virus may be transmitted to the inner ear through cerebrospinal fluid, the nose, and olfactory foramina to the central nervous system; through the endolymphatic sac, through labyrinthine artery to stria vascularis. The damage of the audio-vestibular system during a SARS-CoV2 infection could be related to a direct impairment of inner ear structures or to a virus-mediated immune response. Blood vessels, lymphatics, and nerves and in some cases the meninges have been proposed as entry routes for the virus. The virus can pass through the Eustachian tube or external auditory canal, middle ear, and mastoid to round or oval windows [13]. SARS-CoV-2 is thought to cause an inflammatory response and increase in cytokines, which causes inflammatory response in the cochlea causing cochleitis or neuritis. The virus can also induce coagulopathic and inflammatory edema leads to cochlear ischemia [14]. The inner ear structures are particularly susceptible to ischemia as they are terminal vasculature with high-energy requirement. Both primary and secondary vasculitis are commonly associated with audiovestibular symptom in COVID-19. Cross-reactions of the antibodies or T cells to the inner ear antigens as a result of viral infection leading to accidental damage to the inner ear. Vascular disorders may be also a possible cause of AV manifestation as the cochlea and semicircular canals have no collateral blood supply and are largely susceptible to ischemia [15]. The immune system had been also proposed to be involved in COVID-19 such as over-production of proinflammatory cytokines which have adverse effect on the audio-vestibular system [16].

Harenberg et al. [17] hypothesized that hearing loss in COVID-19 patients may be a sequence of endothelial damage and microthrombi at the central auditory pathway including the brainstem, the auditory nerve, or the cochlea. Generally, coronaviruses (including SARS-Cov-2 virus) have neuro-invasive characteristics and can induce sensory neuropathy [18]. COVID-19 was reported to be associated with Guillain Barre Syndrome (GBS), in some cases which is an acute immune-mediated disease with central and peripheral nerve manifestations [19].

### Clinical presentation

The symptoms and severity of COVID-19 vary from asymptomatic to severe or fatal. The UK National Institute for Health and Care Excellence (NICE, 2020) [20] has provided three phases of signs and symptoms of COVID-19:

- Acute, persisting for up to 4 weeks
- Ongoing, from 4 to 12 weeks.
- Post-COVID syndrome, continuing for more than 12 weeks.

Phases b and c are often grouped together and referred to as long COVID with common symptoms of dizziness, tinnitus, and otalgia [20].

SARS-CoV-2 is characterized by its infectious abilities. Viral pneumonia is the most frequent serious clinical picture of COVID-19 with sever manifestation such as fever, dry cough, dyspnea, hypoxemia, and bilateral infiltrates on chest radiography in addition to fatigue, shortness of breath, anosmia, and loss of taste. However, some cases had no symptoms [21]. A wide range of otorhinolaryngological symptoms had been identified with COVID-19 (Table 1).

**Table 1:** Summary of the otolaryngological manifestations of COVID-19

Otolaryngological manifestations	
***Hearing loss***	Sudden SNHL High-frequency SNHL Auditory neuropathy
***Vestibular manifestations***	Vertigo Dizziness
***Tinnitus***	Roaring noise or high frequency tones Unilateral or bilateral
***Other manifestations***	Facial palsy Acute otitis media Otalgia Otitis externa Migraine

#### Hearing loss

The estimated prevalence of hearing loss in COVID-19 infection is about 7.6% which should be interpreted with caution as most studies used medical records or self-reported symptoms through telephone call or questionnaires without appropriate audio-vestibular evaluation due to home isolation of patients [22, 23]. Jeong et al. [13] reported that HL range from mild to profound. De Sousa et al. [24] reported elevated hearing thresholds at 1000, 2000, 4000, and 8000Hz in patients with mild COVID-19 symptoms. They also reported that the hearing thresholds were worsened in patients with moderate-severe forms of the disease. Other authors confirmed the association between hearing deterioration and the severity of SARS-CoV-2 [25].

Several authors reported sudden SNHL (SSNHL) in COVID-19 patients without prior otologic problems or use of ototoxic medication [16, 25–27]. Regarding the possible mechanism of HL in COVID-19 patients, Cure and Cure [28] suggested the binding of the SARS-CoV-2 virus to the ACE2 which has high level in the central auditory areas in the temporal lobe. This increases the viral load in the tissue induces the release of cytokine followed by oxidative stress condition and irreversible HL [29]. Tissue hypoxia occurs as a result of low oxygen release from the RBCs with further cellular damage. The presence of ACE2 in the vascular smooth muscles made SAR-Cov-2 virus to increase the risk of thrombosis. Hearing loss is probably irreversible as it involves damage to central auditory structures secondary to ischemia or to thrombosis [28]. Satar [29] proposed that the relationship between HL (after careful exclusion of other causes of HL) and COVID–19 could be confirmed if HL occur in patients with confirmed COVID-19 diagnosis using PCR or IgM antibody titers or pulmonary CT scan. The onset of HL should occur within 3 or 4 weeks of COVID-19 infection in association with some vestibular symptoms.

The elderly people have high incidence of comorbidity such as hypertension, atherosclerosis, and diabetes mellitus which increase their risk for thrombosis and the occurrence of HL with their COVID-19 infection [28]. In an interesting finding, Mustafa [30] reported significantly elevated high-frequency pure-tone thresholds in asymptomatic cases in addition to the reduced amplitudes of transient evoked otoacoustic emissions (TEOAEs) when compared with non-infected subjects. More recently, in their series, Jeong et al. [13] reported absent OAEs in COVID-19 patients suggesting OHCs damage in COVID-19 disease

The neurotrophic and neuro-invasive characteristics of SAR-Cov-2 virus can lead to peripheral neuropathy including the auditory neuropathy (AN). Sedaghat and Karimi [31] reported that COVID-19 is associated with Guillain Barre Syndrome (GBS), an acute immune-mediated disease with central and peripheral nerves manifestations.

#### Vestibular manifestation

Vertigo or dizziness has recently been described as a clinical manifestation of neuro-invasive characteristics of COVID-19 as a result of acute labyrinthitis, vestibular neuritis, acute otitis media, or secondary to stroke following COVID-19 [32].

Viola et al. [33] reported balance disorders in 18.4% of their cases after COVID-19 diagnosis with 94.1% reported dizziness and 5.9% reported acute vertigo attacks. Benign paroxysmal positional vertigo has been clinically reported in few COVID-19 patients. It could be related to prolonged hospitalization and bed rest that may contribute to otolith detachment. Vestibular neuritis had been reported with COVID-19; however, lack of vestibular evaluation of COVID-19 cases limited the certainty of the diagnosis in many cases [33].

Uranaka et al. [34] reported the presence of ACE2 receptors in the Eustachian tube, the middle ear and in the inner ear which act as a gate for the SARS-CoV-2 to enter the vestibule and lead to vestibular neuritis. Reactivation of herpes simplex virus (HSV-1), hypoxia, hypercoagulopathy, and immune-mediated insult were among the postulated mechanism of neuro-invasion leading to dizziness. Direct central nervous system insult or vascular damage (vasculitis or vasculopathy) are also possible mechanisms [35].

#### Tinnitus

The data of tinnitus in COVID-19 were collected using self-reported questionnaires such as Tinnitus Handicap Inventory [36]. Through an online 10-item close-ended questionnaire, Viola et al. [33] reported the presence of tinnitus in 23.2% in patients with a positive nasopharyngeal swab for SARS-CoV-2. The neurotrophic and neuroinvasive capabilities of the coronaviruses could be responsible for tinnitus [26]. Tinnitus character and course in COVID-19 were unclear in many studies. However, some studies reported that tinnitus was roaring noise or high frequency tones [26]. Regarding the laterality, it could be bilateral [16], right-sided or left-sided [37], and intermittent or continuous [33]. Variable characters include non-pulsatile [37], disabling [27], or gradually worsening. In many cases, tinnitus had been reported as a new complaint or as worsening of an already existing one [25, 26, 38]. The restrictive measures used to reduce social interactions had a negative impact on wellbeing and increased mental health difficulties specially tinnitus patients leading to its aggravation or worsening.

Regarding the tinnitus onset, there is a lack of consistency where it could be reported with the diagnosis of COVID-19 [37], after discharge from the intensive care unit (ICU) and recovery [39, 40], 1 week after recovery or after recovery in association with HL. The data of tinnitus duration are lacking where Maharaj and Hari [41] reported recovery of tinnitus occurred 2 months after recovery from COVID-19, while Cui et al. [38] reported alleviation of tinnitus and dizziness after treatments with anti-vertiginous drugs (beta-histine). At the contrary, Chirakkal et al. [26] and Lamounier et al. [27] reported tinnitus to persist post-recovery.

#### Facial palsy

The neuro-invasive properties of coronaviruses are responsible for acute facial palsy in COVID-19 patients either isolated or unilateral [42] or bilateral in the context of Guillain–Barre syndrome [43]. Tamaki et al. [44] reported 0.08% incidence of facial palsy in COVID-19 patients which increase to 8.6% in patients with previous history of facial palsy. Islamoglu et al. [45] reported positive SARS-CoV-2 IgG and IgM in 24.3% of facial palsy patients who are asymptomatic or had no history of COVID-19. Additionally, Codeluppi et al. [46] reported that 21% of patients presenting to the emergency department for facial palsy during the outbreak had symptoms consistent with COVID-19 infection. The mechanism of facial palsy in COVID-19 may be related to direct viral neuro-invasion or immune-mediated mechanism secondary to increased pro-inflammatory cytokines causing damage the neuronal tissues [44].

#### Acute otitis media

Exposure to viruses may lead to upper respiratory tract infection with mucus secretions and damage to the ciliated columnar epithelium in the middle ear, Eustachian tubes, and sinuses. Fidan [47] reported a case of PCR-positive of COVID-19 with clinical picture of acute otitis media (AOM) presented only with otalgia and tinnitus. Otoscopy revealed right red bulging tympanic membrane, and audiologic evaluation showed right conductive hearing loss with type B tympanogram. To confirm the diagnosis of COVID-19-induced otitis media, sampling of the middle ear should be done [48].

#### Otitis externa

Mady et al. [49] reported 18% incidence of otitis externa (OE) in COVID-19 patients. The pathogenesis of OE might be due to immune response or the presence of ACE2 in the skin which is the main receptor of SARS-CoV-2 virus [49].

#### Otalgia or earache

Otalgia was reported in case reports and cross-sectional studies in COVID-19 patients. This symptom was attributed mainly to acute otitis media and otitis externa in those patients. The incidence is unknown due to small sample size of studies, however. Earache could be the clinical symptoms during the early phase of the coronavirus infection [50].

#### Migraine

There is an association between otoneurological manifestation of COVID-19 and migraine which was present in 30.8% of COVID-19 cases studied by Viola et al. [33]. Those authors reported that 5.9% of tinnitus patients and 7% of patients with equilibrium disorders were also affected by migraine. Moreover, 2.7% of patients complaining of both tinnitus and balance disorders after COVID-19 diagnosis were also affected by migraine. The pathobiology of migraine in COVID 19 patients actually is not clear and were postulated possibly a result of the body’s inflammatory response to the virus [33].

### COVID-19 medical treatment and ototoxic manifestations

Many of previously or currently used drugs in treating COVID-19 have known ototoxicity (Table 2). These drugs include the antimalarials, immunomodulatory agents, macrolide antibiotics, and antiviral drugs. Ototoxic drugs can induce cellular damage to tissues of the inner ear including both the cochlear and vestibular system with subsequent hearing loss, tinnitus, and/or disequilibrium [51]. These symptoms could be permanent if not recognized early. For instance, hydroxychloroquine and chloroquine prescribed for almost 12% of COVID-19 patients in Europe had faster virological clearance but high-quality evidence of their potential benefit still not proven yet [52]. However, they have known adverse events on the inner ear, including tinnitus and hearing loss [53]. They also might induce balance disorder and the symptoms may be misdiagnosed as being caused by COVID-19. The characteristics of SNHL and/or tinnitus after chloroquine or hydroxychloroquine treatment can be temporary but reports of persistent auditory and vestibular dysfunction exist [54].

**Table 2:** The common drugs used in treatment of COVID-19

Drug	Category
Hydroxychloroquine, chloroquine	Antimalaria
Azithromycin	Macrolide antibiotic
Interferons (IFNs)	Antiviral and immunoregulatory agents
Lopinavir, ritonavir, ribavirin	Antiviral
Ivermectin	Antiparasitic

Other types of treatment protocols used multiple drugs for COVID-19 including *Azithromycin* which is a macrolide antibiotic with known anti-inflammatory properties and inhibits viral replication of human influenza virus H1N1 in vivo. It is documented that azithromycin can cause HL, tinnitus, and imbalance. HL is usually irreversible and range from mild to severe SNHL even at standard oral doses of 250 mg [55]. Although the drug is incorporated in many COVID-19 treatment protocols, its efficacy is not yet proven [56].

Interferons (IFNs) are natural antiviral and immunoregulatory agents that react to viral infections and determine the immune response to the viral infection. In COVID-19 disease, IFN-α therapy significantly reduced rates of viral shedding and levels of inflammatory markers, whereas IFN-β therapy improved virologic clearance [57]. Accordingly, both IFN-β and IFN-α were used in COVID-19 patients. However, these drugs have serious ototoxic effects including hearing loss and tinnitus either due to direct ototoxicity with high-dose interferon, autoimmune-mediated microvascular damage, or hematological changes. HL is generally reversable and returned to normal within 14 days of discontinuing therapy [58]. In COVID-19, aging and male gender have been identified as risk factors for increased severity of the disease and raise the concern for increased risk of IFN-induced ototoxicity in these already vulnerable populations [59].

Some antiviral drugs are listed among current treatments against SARS-CoV-2 including the drug combination of *lopinavir–ritonavir* (antiviral protease inhibitor). Few reports described anecdotal cases of hearing loss associated with their use, postulating possible mitochondrial toxicity as a potential etiologic mechanism. Moderate, bilateral HL was confirmed by audiologic testing in a patient 4 weeks after initiation of lopinavir-ritonavir therapy which was reversible after discontinuation of treatment [60]. *Ribavirin* is another antiviral agent that interferes with viral replication through inhibition of viral mRNA synthesis. Minimal data exists on the ototoxic effects of ribavirin alone as it is usually combined with INF for treating COVID-19 patients. Data from several case report studies showed that this combination was followed by HL which could be unilateral or bilateral, permanent, or reversible in addition to tinnitus [61]. Interestingly, the studies showed that HL induced by IFN therapy alone is usually reversible; however, that rate of irreversible HL is increased with the IFN/ribavirin combination therapy suggesting that ribavirin may enhance known ototoxic effects [59].

*Ivermectin* is a broad spectrum antiparasitic drug that has been used recently as a treatment of COVID-19 due to its inhibitory effect on the replication of the SARS-CoV-2 virus in vitro [62]. It is used in combination with other drugs (azithromycin, chloroquine, hydroxychloroquine, and ribavirin). Generally, its ototoxic effects appear in the form of disequilibrium that was self-limited [59].

Most drugs used in the treatment of COVID-19 are usually administrated in combination and all of them have ototoxic effects. The condition is worsened additional factors like aging, underlying hearing impairment, comorbidity, genetic factors, and renal impairment. Renal impairment occurred in 20 to 40% of critically ill COVID-19 patients, and this will reduce the rate of elimination of ototoxic drugs from the body and increase the risk of ototoxicity. Additionally, hypoxia (which is a defining feature of serious COVID-19 disease) and the hypercoagulable state associated with COVID-19 may exacerbate the ototoxic effects of drugs and increase the risk of HL due to microthrombi formation in these patients [59].

So, the American Speech–Language–Hearing Association (ASHA, 1994) guidelines are used for all patients with COVID-19 who should be audiologically monitored during the treatment course, receive post treatment audiologic evaluations at 3 months and 6 months after cessation of treatment, and receive prompt aural rehabilitation if ototoxic effects should manifest [59].

#### Vaccination and audiovestibular manifestation

Different vaccines were used in COVID-19 disease such as BNT162b2 mRNA Pfizer-BioNTech COVID-19 and the mRNA-1273 SARS-CoV-2Moderna COVID-19. During the early release of the vaccines, otologic symptoms were not listed as common potential adverse effects. But later, there was an increase in the frequency of patients presenting with SSNHL, tinnitus, aural fullness, and/or the exacerbation of previously stable Menière’s in addition to autoimmune inner ear disease (AIED) [63].

The House Ear Clinic developed a study in subjects within 30 days after they received COVID-19 vaccination. The study was conducted along 3 years (2019, 2020. and 2021). They reported an increase in the incidence of SSNHL from 1.60% in 2019 to 2.44 and 3.85% in 2020 and 2021, respectively. They also reported that hearing loss as the most common symptom followed by tinnitus, dizziness, and aural fullness. The mean time to onset of symptoms was 10.2 ± 9  days after vaccination [64]. In another study, Almufarrij et al. [23] reported a prevalence of 7.6% for hearing loss, 14.8% for tinnitus, and 7.2% for rotatory vertigo. The possible causes of post-vaccination otological manifestation could be the immunological response that may cause vasculitis and ischemia of the cochlea [64]. Another hypothesis is that the mRNA vaccines (like Pfizer and Moderna vaccines) can induce a possible reactivation of previous latent viruses resulting in SSNHL or facial Palsy [65].

The exacerbation of AIED diseases including Meniere’s were noted after the first COVID-19 vaccination with further decrease of hearing noticed at the time of follow-up following the second COVID vaccine dose [66]. Abouzari et al. [67] highlighted the common link between post-vaccination migraines and hearing loss. They noticed an increase of headache symptoms in 51% of cases after the Pfizer vaccine second dose in older patients. Additionally, headaches were reported to be the most common side effect after the second dose of Moderna vaccine [67]. The exact link between post vaccination migraine and hearing loss is not clear and were unexplained, and further researches regarding this point are required.

## Conclusion

There is growing evidence that audiovestibular symptoms may represent atypical manifestation of COVID-19 or even the only presentation so COVID-19 should be taken in consideration in patients presented with audiovestibular manifestations for early diagnosis and better prognosis and also decrease the potential infection risks to ENT specialists. The exact pathogenesis of audiovestibular manifestations in COVID-19 patients is not well known. Audiovestibular manifestations may be sequalae of the SARS-CoV-2 virus or as side effects of the ant COVID-19 medications; these manifestations may be reversible or not according to the severity of the condition. There is an increase in frequency of the audiovestibular symptoms after COVID-19 vaccinations. More large-scaled studies are needed to understand the pathogenesis and the audiovestibular complications, the risk factors for developing this challenging disease, and audiovestibular manifestations after vaccination and the relation to different medications.

## Data Availability

Not applicable
